# Enhanced anticancer activity of silver doped zinc oxide magnetic nanocarrier loaded with sorafenib for hepatocellular carcinoma treatment

**DOI:** 10.1038/s41598-024-65235-6

**Published:** 2024-07-05

**Authors:** Doaa S. R. Khafaga, M. M. Eid, Mona H. Mohamed, Mohamed D. E. Abdelmaksoud, Mie Afify, Ahmed M. El-Khawaga, Heba K. Abdelhakim

**Affiliations:** 1 Biochemistry Division, Faculty of Science, Cairo University, Giza, 12613 Egypt; 2 Department of Basic Medical Sciences, Faculty of Medicine, Galala University, New Galala City, Suez, 43511 Egypt; 3 Spectroscopy Department, National Research Centre, Dokki, Cairo, Egypt; 4 Department of Chemistry, Faculty of Science, Cairo University, Giza, 12613 Egypt; 5 Biochemistry Department, Biotechnology Research Institute, National Research Centre, Cairo, Egypt

**Keywords:** Zinc oxide, Hepatocellular carcinoma, Diethyl nitrosamine, Sorafenib, Nanocomposite, Drug delivery, Drug delivery, Cancer therapy, Nanomedicine

## Abstract

Drug delivery is the process or method of delivering a pharmacological product to have therapeutic effects on humans or animals. The use of nanoparticles to deliver medications to cells is driving the present surge in interest in improving human health. Green nanodrug delivery methods are based on chemical processes that are acceptable for the environment or that use natural biomaterials such as plant extracts and microorganisms. In this study, zinc oxide-superparamagnetic iron oxide-silver nanocomposite was synthesized via green synthesis method using *Fusarium oxysporum* fungi mycelia then loaded with sorafenib drug. The synthesized nanocomposites were characterized by UV-visibile spectroscopy, FTIR, TEM and SEM techniques. Sorafenib is a cancer treatment and is also known by its brand name, Nexavar. Sorafenib is the only systemic medication available in the world to treat hepatocellular carcinoma. Sorafenib, like many other chemotherapeutics, has side effects that restrict its effectiveness, including toxicity, nausea, mucositis, hypertension, alopecia, and hand-foot skin reaction. In our study, 40 male albino rats were given a single dose of diethyl nitrosamine (DEN) 60 mg/kg b.wt., followed by carbon tetrachloride 2 ml/kg b.wt. twice a week for one month. The aim of our study is using the zinc oxide-superparamagnetic iron oxide-silver nanocomposite that was synthesized by *Fusarium oxysporum* fungi mycelia as nanocarrier for enhancement the sorafenib anticancer effect.

## Introduction

Engineered nanoparticles (NPs) are utilized in nanomedicine for treating diseases, which is a biological application of nanotechnology. Nanomedicine has the potential to help in the early identification and treatment of cancer because of its cutting-edge imaging and therapeutic capabilities^1^. Active or passive targeting, high solubility, biocompatibility, bioavailability, and multifunctionality are all advantages that make it superior to conventional cancer therapies. Green synthesis techniques can produce environmentally beneficial nanoparticles^2,3^. Consequently, using green technology is becoming more common than ever^4^. The nanoparticles act as biomaterials to deliver drugs^5,6^. Single-drug chemotherapy, often known as monotherapy, is insufficient to give a full therapeutic strategy for cancer treatment. As a result, efforts have been focused on developing drug delivery systems that co-deliver numerous combinations of therapeutic agents to overcome the limitations of single-drug chemotherapy. Low response rates, inefficiency in completing tumor treatment, and drug resistance are only a few of the drawbacks. These disadvantages result in tumor recurrence and aggressive proliferation^7,8^. Hepatocellular carcinoma (HCC) accounts for over 90% of all primary liver cancer cases^9^. For the treatment of individuals with advanced hepatocellular carcinoma, the US Food and Drug Administration authorized the tyrosine kinase inhibitor sorafenib in 2007. Endothelial cells and pericytes' vascular endothelial growth factor receptors (VEGFR) and platelet-derived growth factor receptors (PDGFR) can be targeted for reducing angiogenesis, whereas tumor cells' B-RAF and RAF1 can be inhibited to reduce cell proliferation. However, a greater percentage of the sorafenib dosage for oral administration in pharmaceutical form is lost due to oxidation or glucuronidation, reducing its efficacy and potency in the treatment of HCC patients^10^. In addition, unpleasant side effects of sorafenib include diarrhea, hypertension, an acneiform rash, and weariness. Trans arterial chemoembolization or surgery moreover to sorafenib does not significantly increase overall survival. Only 30% of individuals with advanced HCC can benefit from sorafenib before developing medication resistance and unfavorable side effects^11^. Since the biopharmaceutical classification system (BCS) enlists sorafenib as a class II drug, due to its low solubility and permeability features and solubility over a varied pH range (1.2–7.4), it dissolves sluggishly in the gastrointestinal tract (GIT), which is considered an absorption rate-limiting step and because of its first-pass mechanism, which leads to reduced bioavailability and large inter-subject variations. Liver enzymes (predominantly CYP3A4) metabolize sorafenib and excrete it through the bile duct; urinary excretion also accounts for a minor (less than 20% route of elimination)^12,13^. So numerous approaches for formulation have been used to advance the physiochemical limitations and raise the bioavailability of sorafenib^14^.

Nanotechnology has shown its application in effective drug delivery to target sites using diverse anti-cancer agents. Using oral, intranasal, intra-venus, and transdermal routes These nanocarriers have been widely dynamic to improve the absorption of drugs that are poorly soluble in water^15,16^. Several records considered sorafenib-loaded nanoparticles to have a valuable effect on both HepG2 cells in vitro and in a DEN-induced HCC rat model in vivo, with a distinguished advantage over the conventional sorafenib itself^17^. These auspicious outcomes may shed innovative light on a novel therapeutic approach for patients with HCC. So co-delivery of sorafenib, in its newly nano-designed formula would suggest another anti-cancer drug which could be the next step to further enhance their therapeutic efficacy to efficiently fight HCC^18^.

Our aim of study is the biological production of superparamagnetic iron oxide-silver nanoparticles (SPION-Ag NPs) utilizing *Fusarium oxysporum* fungus and modification with zinc oxide nanoparticles (ZnO NPs) to be used as a drug delivery system to enhance the anticancer effect of sorafenib, avoid its side effects and studying the expression of some genes related to mechanism of cancer in liver cells in diethylnitrosamine-indued hepatocellular carcinoma in rat model.

## Materials and methods

### Chemicals

Iron (III) nitrate (Fe(NO_3_)_3_), ammonium iron (II) sulfate hexahydrate ((NH_4_)_2_Fe(SO_4_)_2_⋅6H_2_O) and silver nitrate (AgNO_3_) were purchased from Merck (Germany). Sodium hydroxide and zinc acetate were purchased from Sigma Aldrich. Sorafenib (formerly Nexavar^®^) was generously supplied by Bayer AG of Germany. Carbon tetrachloride (CCl_4_) was purchased from Al-Gomhoria Company (Cairo, Egypt). Diethyl nitrosamine was purchased from Gene Tech Company. Sodium pentobarbital as an intraperitoneal (IP) anaesthesia was purchased from Sigma Aldrich. All other reagents were analytical grade and directly used without any purification.

### Loading of the sorafenib on ZnO-SPION-Ag nanocomposite

ZnO-SPION-Ag nanocomposite was synthesized according to Doaa S. R. Khafaga et al.^1^ using *Fusarium oxysporum* fungus as green synthesis method. An equal amount of ZnO-SPION-Ag nanocomposite and sorafenib drug were grinded then dissolved in 25 ml sterile distilled water and sonicated at different time to select the best time for allocation of the drug on the nanoparticles and dried at 40 °C overnight.

### Experimental design

A total of 40 male albino rats weighing between 76 and 130 g were obtained from the National Research Centre (NRC) in Dokki, Egypt. The rats were housed in suitable plastic enclosures, with each cage accommodating 8 rats and equipped with stainless steel wire lids. The rats were kept in typical laboratory settings, following a 12-h cycle of light and darkness. They were provided with a conventional diet and access to water. The animal procedures were conducted in accordance with the recommended requirements for experimental animal care guidelines. Also, the study is reported in accordance with ARRIVE guidelines, and this study was approved by the ethics committee of the institutional animal care and use committee (CU-IACUC), Cairo University under (Approval no. CU/I/F/100/17). The rats were allocated randomly into five groups, with each group consisting of 8 rats. Group (I): The healthy group designated as the negative control. Group (II): Rats were intraperitoneally administered with diethyl nitrosamine at a single dose of 60 mg/kg b.wt. This was followed by administration of CCl_4_ at a dose of 2 ml/kg b.wt. twice a week for one month. The purpose of this treatment was to develop hepatocellular carcinoma and this group served as the positive control^19^. Group (III): HCC was induced group treated by sorafenib 10 mg/kg b.wt. for 15 days^20^. Group (IV): HCC was induced group treated by ZnO-SPION-Ag nanocomposite 10 mg/kg b.wt. for 15 days. Group (V): HCC was induced group treated by ZnO-SPION-Ag nanocomposite loaded with sorafenib 10 mg/kg b.wt. for 15 days. Rats had been starving overnight at the end of the experiment. Blood samples were obtained 15 days after treatment while being mildly sedated with 40 mg/kg sodium pentobarbital as an intraperitoneal anaesthesia using retro orbital puncture technique, samples were kept at room temperature for 15 min, and centrifuged for 15 min at 4000 rpm for obtaining serum. For biochemical analysis, serum samples were kept at − 20 °C. Rats underwent cervical dislocation after blood samples were taken, livers were removed immediately, washed in ice-cold normal saline solution 0.9%, and dried with filter paper to remove excess saline^21^. A weighted portion of each rats livers left lobe was removed and stored in 10% formalin for histopathological examination, and another weighted portion was frozen at − 80 °C for molecular and antioxidants analysis. The liver index was calculated after the animals were sacrificed as: Liver weight (g)/Final body weight (g) × 100.

### Biomarker genes for HCC

#### Isolation of RNA

The total genomic RNA in the liver tissues of all treated animals was isolated using TRIzol^®^ extraction Chemical (Invitrogen). Following the completion of the isolation procedures, the RNA pellet was kept in DEPC treated water. The isolated RNA pellet was treated using an RNAse-free DNAse kit (Invitrogen, Germany) to digest the possible DNA residues. RNA aliquots were either stored at − 20 °C or used for reverse transcription immediately^22^.

#### Reverse transcription reaction

The reverse transcription process (RT) was utilized to generate the cDNA copy from liver tissues using the First Strand cDNA Synthesis Kit (RevertAidTM, MBI Fermentas). To acquire the cDNA copy of the liver genome, RT reaction schedule of 25 °C for 10 min, 1 h at 42 °C, and 5 min at 95 °C was employed. Finally, tubes of reaction containing cDNA copies were placed on ice to be used for cDNA amplification^23^.

#### Quantitative real time-PCR

The qRT-PCR studies were carried out using the SYBR® Premix Ex TaqTM kit (TaKaRa, Biotech. Co. Ltd.) and generated cDNA copies from liver tissues. A melting curve profile was created for each reaction. Table 1 illustrates how the quantitative values of the target genes were normalized based on the expression of the housekeeping gene. The quantitative values of the individual genes were determined using the 2^−ΔΔCT^ technique in comparison to the β-actin gene.

**Table 1:** qPCR primer sequences utilized: F (forward primer) and R (reverse primer).

Gene	Primer sequence (5–3ʹ)	References
DTL	F-CCT CTG TCC GAT CCT CCA AA	^24^
	R-AAA GAT TTT CAG TCC CGC GG	
DUSP1	F-AGA ATG TTC CTG ACT CGG CA	^24^
	R-AAG CAA AAT CCA ATC CCG GG	
NFKBIA	F-AGC GAT GGG GTC TCA CTA TG	^24^
	R-TCC AAC AGC TTA GGT CAG GG	
SOCS2	F-GCT TGG GGT TAA ATG GTG CA	^24^
	R-AAG GGA TGG GGC TCT TTC TC	
β-actin	F-GGT ATG GAA TCC TGT GGC ATC CAT GAA A R-GTG TAA AAC GCA GCT CAG TAA CAG TCC G	^25^

Genes targeted: (DTL) denticleless protein homolog, (DUSP1) dual specificity phosphatase 1, (NFKBIA) nuclear factor of k light chain gene enhancer in B cells inhibitor, α, and (SOCS2) suppressor of cytokine signaling 2.

### Biochemical analysis

The biochemical markers used to assess early liver injury were serum ALT, AST^26^ and ALP^27^ activates and bilirubin, which were measured using quantitative colorimetric commercial kits (Biodiagnostic). The activity of tissue liver enzymes GST^28^ and GPx^29^ were measured using quantitative colorimetric kits (Biodiagnostic). The estimation of lipid peroxidation (MDA) was conducted using the method developed by Ohkawa^30^ and NO according to Sayed D^31^. The measurement of catalase activity was conducted using the Aebi method^32^. The measurement of superoxide dismutase activity was conducted using the Nishikimi et al. method^33^. The concentration of serum alpha-fetoprotein (AFP) was measured using ELISA Biocheck kits (USA)^34^.

### Histopathological examination

Histopathological analysis was done according to the method was mentioned in Doaa S. R. Khafaga et al. at the end of study, animals were sacrificed after heavy anesthesia, rapidly dissected, and small slices of the liver were instantly taken for fixation in 10% formalin for 24 h. The samples underwent a series of steps, including washing in tap water, dehydration using increasing concentrations of ethanol, clearing in xylene, and finally embedding in paraffin wax with a melting point of 56–60 °C. Tissue sections with a thickness of 6 µm were produced and treated with haematoxylin and eosin stain. The paraffin sections were stained with Harris haematoxylin for a duration of 5 min using this technique. The sections were rinsed in flowing water to enhance the blue color and then immersed in a 1% aqueous eosin solution for a duration of 2 min. Afterward, they were rinsed with water, dried, clarified, and finally mounted in Canada balsam. The cytoplasm exhibited varying levels of pink to red coloration, whereas the nuclei had a blue color^35^.

### Statistical analysis

Continuous variable data were articulated as mean ± standard deviation (mean ± SD) of 8 animals, and a standard computer software (SPSS for Windows, edition 25.0, IBM SPSS Inc., USA) was utilized for data entry and analysis. The variances through groups were assessed using one-way ANOVA, followed by Turkey's honestly significant difference (HSD) test and a P < 0.05 threshold for statistically significant differences.

### Ethics approval and consent to participate

This study was approved by the ethics committee of the institutional animal care and use committee (CU-IACUC), Cairo University under (Approval no. CU/I/F/100/17).

## Results

### Characterization of the prepared nanocomposites

**Figure 1:**
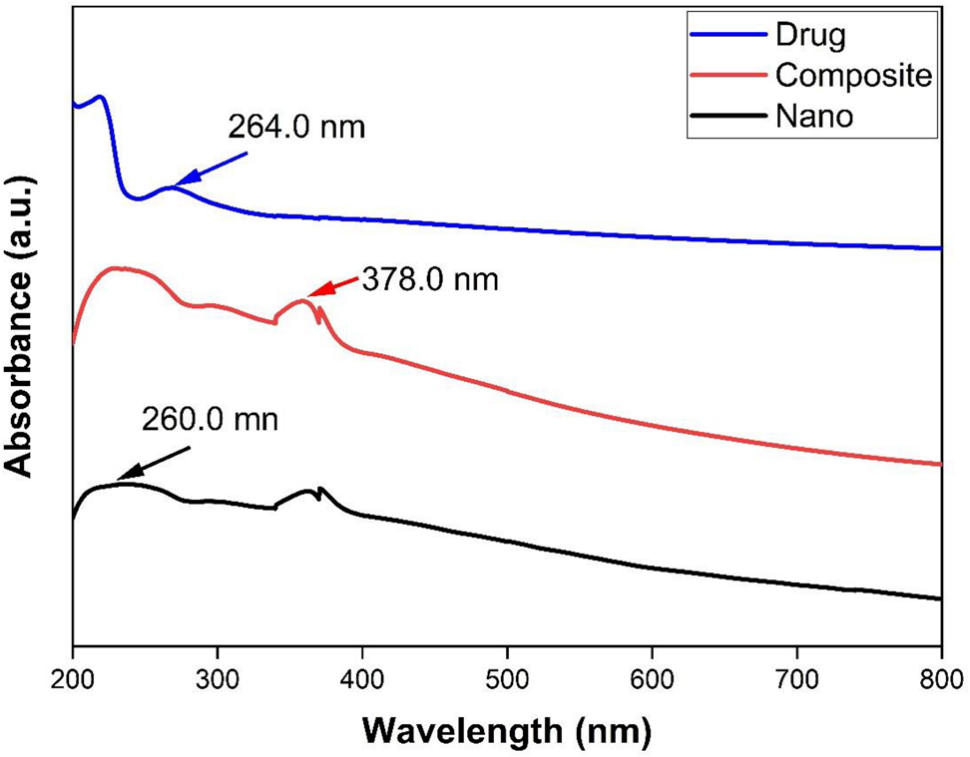
UV–Vis. of ZnO-SPION-Ag NPs (black line), sorafenib (blue line) and sorafenib loaded ZnO-SPION-Ag NPs (red line).

UV–visible absorption spectroscopy is a widely being used technique to examine the optical properties of nanosized particles. The synthesized ZnO-SPION-Ag nanocomposite, ZnO-SPION-Ag nanocomposite loaded with sorafenib and sorafenib drug were characterized by JASCO V-630 UV–Visible (UV–Vis) absorption spectrophotometer has a resolution of 0.2 nm. Figure 1 demonstrated that the weak absorption peak at 378 nm observed in the sorafenib loaded on ZnO-SPION-Ag NPs spectra, was attributed to the plasmonic resonance peak of silver in the composite spectra. The peak found at about 260 nm is mostly because of the ZnO nanoparticles formation in composite sample^36^. Free sorafenib shows a band centered at 264 nm, which belongs to B-band of an aromatic moiety in sorafenib molecule^37^. The interaction of sorafenib drug with the nanoparticles resulted in a blue shift in the characteristic absorption peak of sorafenib at 264 nm and also increasing the intensity of the absorption peak at 378 nm^12^.

**Figure 2:**
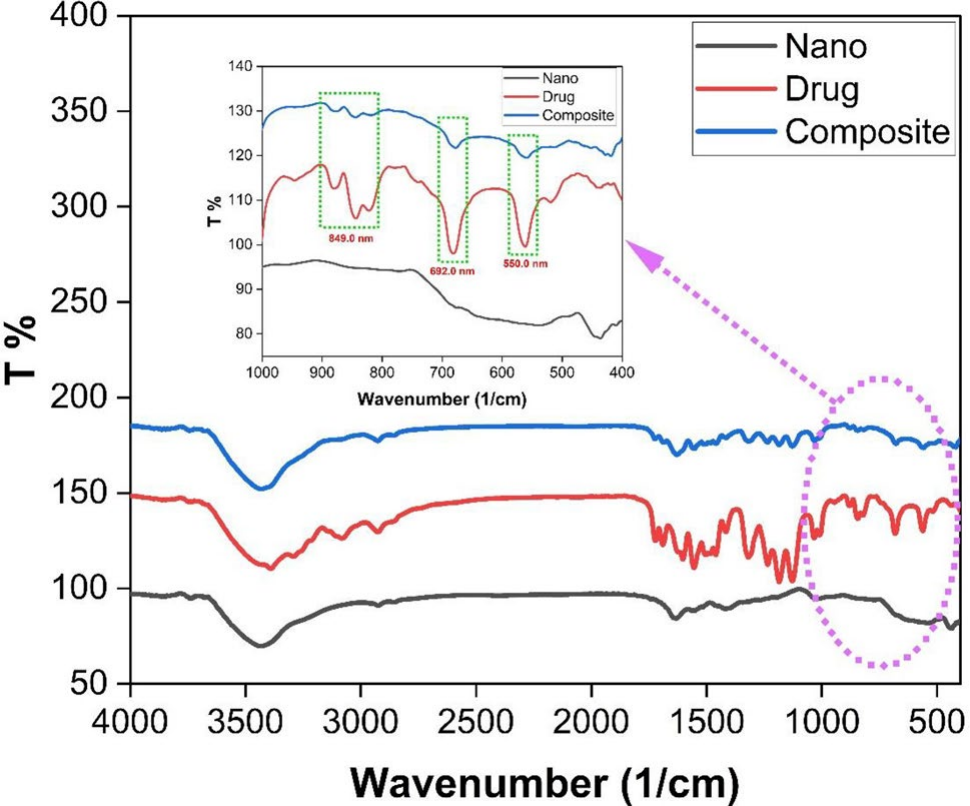
FTIR of ZnO-SPION-Ag NPs (black line), sorafenib (red line) and sorafenib loaded ZnO-SPION-Ag NPs (blue line).

The fourier transform infrared (FTIR) analysis is a suitable method for identifying the specific functional groups present in the organic compounds of the sample. The nanoparticles were analyzed using the KBr-pellet procedure to get their FTIR spectra, which included a range of 500 to 4000 cm^−1^. Figure 2 demonstrated that the green approach for nanoparticle synthesis, as well as the sorafenib structure and sorafenib loading on the nanocomposite, Fourier transform infrared spectroscopy confirms this. The absorption bands at 550 and 450 cm^−1^ related to the vibrations of the Fe–O bond and has thus been used to validate the formation of iron oxide nanoparticles^38–40^. This band confirms the formation of Fe_2_O_3_ as previously demonstrated by Zhang et al.^41^. The presence of these bands shows that superparamagnetic iron oxide nanoparticles are the primary phase of the produced drug-loaded magnetic nanoparticles. The vibrational band for the hydroxy groups was also found in the FTIR spectra, although this was attributed to the samples' significant moisture absorption. The appearance of the three characteristic peaks of sorafenib loaded on ZnO-SPION-Ag NPs at 849, 692, and 550 cm^−1^ and the C=N peak at 1725 cm^−1^ with a significant decrease of the peak at 849 cm^−1^ confirms that the sorafenib was loaded on the nanocomposite.

**Figure 3:**
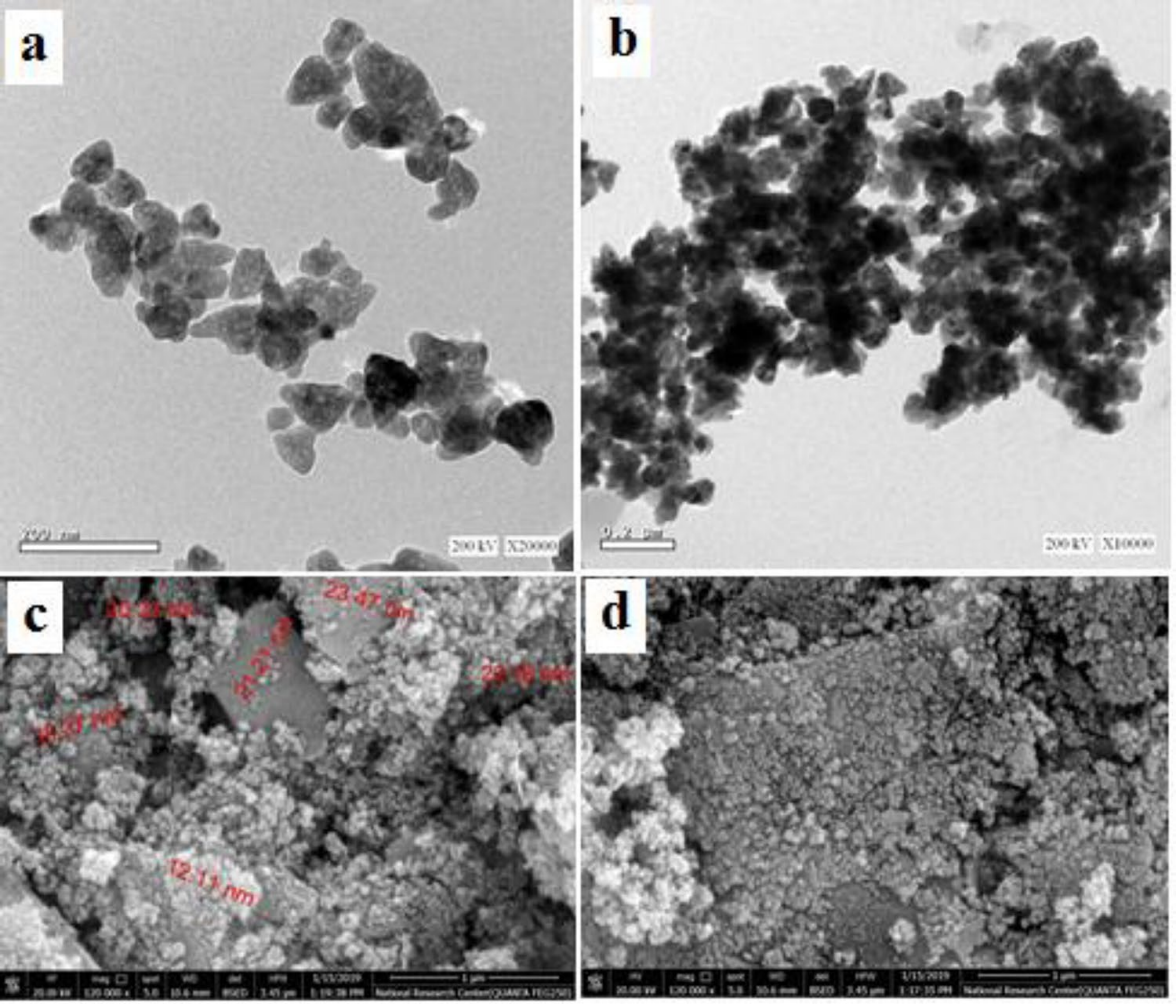
(**a**) The HRTEM of ZnO-SPION-Ag NPs, (**b**) HRTEM of ZnO-SPION-Ag NPs loaded with sorafenib, (**c**) HRSEM of ZnO-SPION-Ag NPs and (**d**) HRSEM of ZnO-SPION-Ag NPs loaded with sorafenib.

To examine the particle size and crystal structure of the sorafenib loaded on ZnO-SPION-Ag NPs, high-resolution transmission electron microscopy (HRTEM) analysis was used. High resolution-Transmission electron microscope HR-TEM (JEM 2100HR, Japan) at 200 keV as shown in Fig. 3. The nanoparticles were single crystal, and because superparamagnetic iron oxide particles become single domain and monopolar at diameters smaller than 50 nm, the sorafenib-loaded magnetic nanoparticles were likewise single domain and monopolar. The SEM photos further indicated that the produced nanoparticles have a spherical shape and near-uniform structures with sizes ranging from 30 to 40 nm.

### Biomarkers genes expression

**Figure 4:**
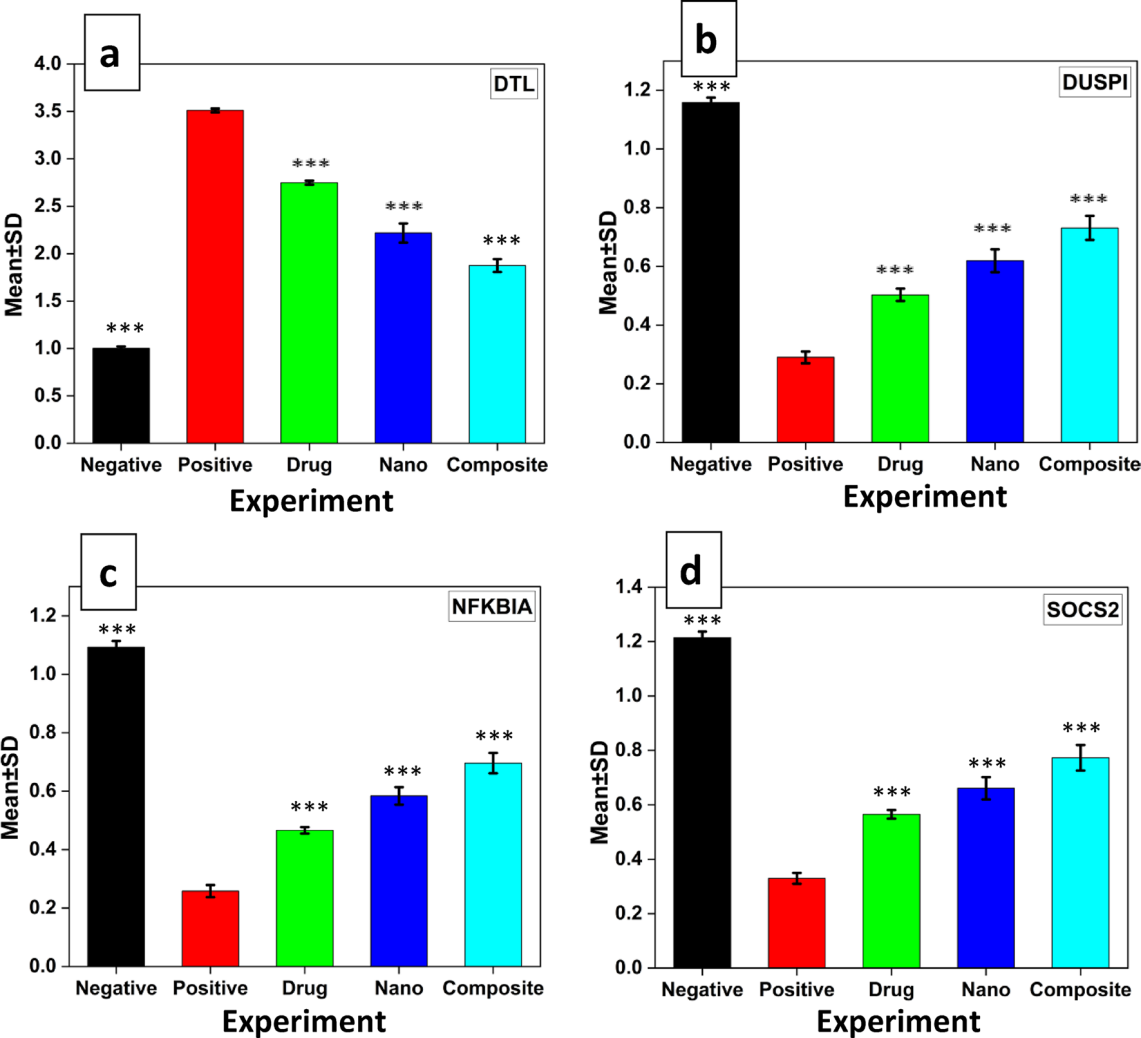
The alterations of DTL-mRNA (**a**), DUSP1-mRNA (**b**), NFKBIA-mRNA (**c**) and SOC2-mRNA (**d**) in liver tissues collected from HCC-induced rats treated with different types of treatments, (black) the negative group, (red) the positive group was induced with HCC, (green) HCC group treated by sorafenib drug, (blue) HCC group treated by ZnO-SPION-Ag nanocomposite, (cyan) HCC group treated by ZnO-SPION-Ag nanocomposite loaded with sorafenib. Three stars (***) mean p-value < 0.001, when compared with the positive control group.

The data illustrated by Fig. 4, show the therapeutic effect of nanocomposite and nanocomposite loaded with sorafenib comparing with the therapeutic effect of standard drug for HCC as sorafenib on genes expression related with HCC disease. There was down-regulation in DTL but up-regulation in other genes. P value < 0.001 between all studied groups as defined by one-way ANOVA test followed by turkey's HSD post-hoc test Fig. 4.

### Biochemical assay

**Figure 5:**
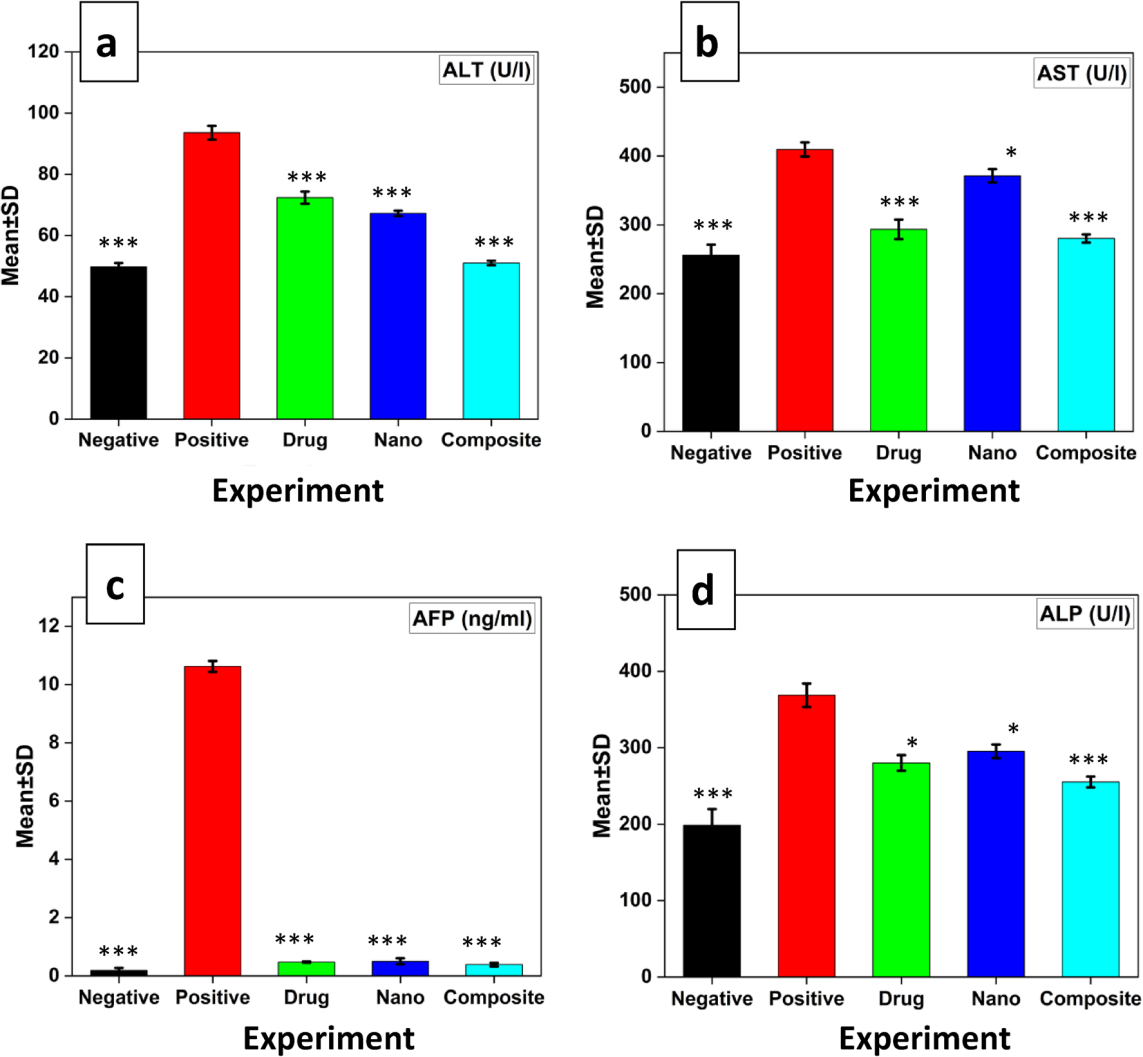
The alterations of serum ALT (**a**), AST (**b**), AFP (**c**), and ALP (**d**); collected from HCC-induced rats treated with different types of treatments at highly significant p value, (black) the negative group, (red) the positive group was induced with HCC, (green) HCC group treated by sorafenib drug, (blue) HCC group treated by ZnO-SPION-Ag nanocomposite, (cyan) HCC group treated by ZnO-SPION-Ag nanocomposite loaded with sorafenib. One star (*) means p-value < 0.05 and three stars (***) mean p-value < 0.001, when compared with the positive control group.

The data expressed by Fig. 5, show the therapeutic effect of nanocomposite and nanocomposite loaded with sorafenib comparing with the therapeutic effect of standard drug for HCC as sorafenib on serum liver function test. There was a highly significant p value < 0.001 increase in comparison between positive control and negative control groups while decrease in compare between all treated and the positive control group (Fig. 5).

**Figure 6:**
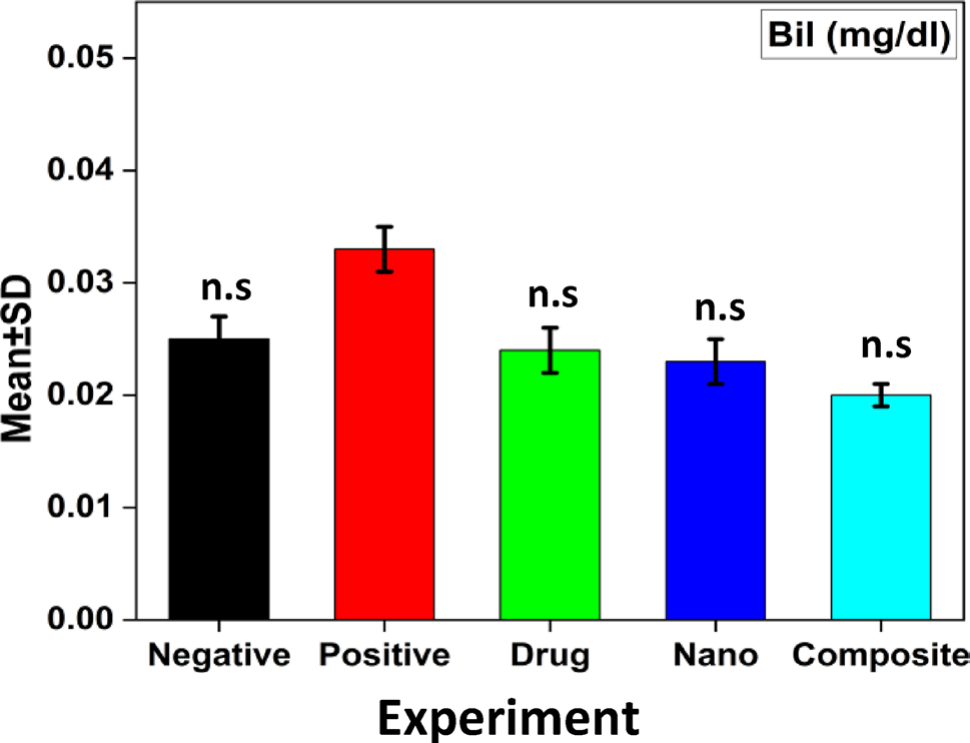
The alterations of serum Bil collected from HCC-induced rats treated with different types of treatments at non-significant (n.s) p value, when compared with the positive control group, **(**black) the negative group, (red) the positive group was induced with HCC, (green) HCC group treated by sorafenib drug, (blue) HCC group treated by ZnO-SPION-Ag nanocomposite, (cyan) HCC group treated by ZnO-SPION-Ag nanocomposite loaded with sorafenib.

**Figure 7:**
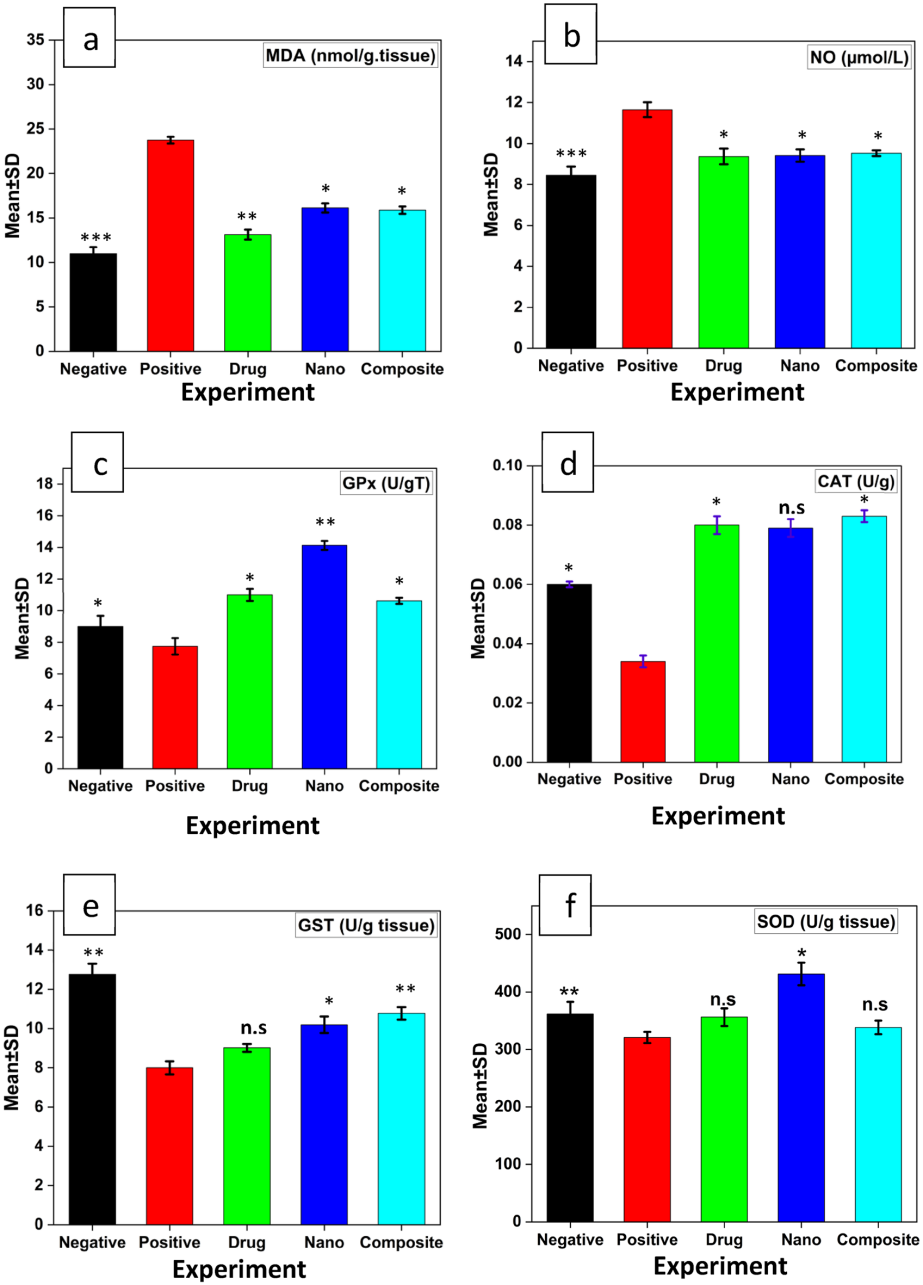
The alterations of tissue MDA (**a**) and NO (**b**) increase in HCC induced group (G II), while GPx (**c**), CAT (**d**), GST (**e**) and SOD **(f)** decrease, (black) the negative group, (red) the positive group was induced with HCC, (green) HCC group treated by sorafenib drug, (blue) HCC group treated by ZnO-SPION-Ag nanocomposite, (cyan) HCC group treated by ZnO-SPION-Ag nanocomposite loaded with sorafenib. One star (*) means p-value < 0.05, two stars (**) mean p-value < 0.01, and n.s means not significant, when compared with the positive control group.

While bilirubin results showed non-significant p value 0.095 increase in compare between positive control and negative control groups while decrease in compare between all treated and the positive control group Fig. 6. Results of MDA and NO non-enzymatic antioxidant showed a highly significant increase p value 0.001 and significant increase p value 0.002 respectively, but results of GPx, CAT, GST and SOD enzymatic antioxidant showed a highly significant decrease p value 0.001 and significant decrease p value 0.019, 0.004 and 0.021 respectively in compare between all studied groups Fig. 7, while MDA and NO decrease and GPx, CAT, GST, and SOD increase in compare between all treated groups and the positive control group.

### Histopathology

**Figure 8:**
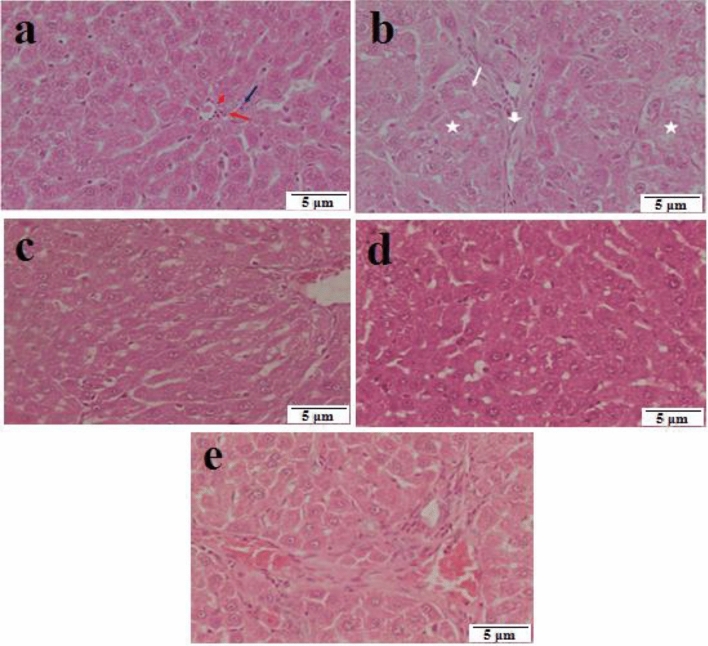
(**a**) This portion displays a typical portal tract from the liver of the negative control group. The components inside the portal tract include the branches of the portal vein (shown by an arrow), the hepatic artery (indicated by an arrowhead), and the bile duct (indicated by a red arrow). (**b**) A micrograph of a rat's liver treated with DEN at a dosage of 60 mg/kg b.wt., followed by CCl_4_ at a dosage of 2 ml/kg b.wt. twice a week for 1 month, reveals the presence of tumour cells resembling pseudo-glands (shown by a white arrow). The presence of fibrosis sheet (shown by an arrowhead) and hydropic degeneration (indicated by an asterisk) is seen. (**c**) A histological image of a rat liver treated with DEN at a dosage of 60 mg/kg b.wt., followed by CCl_4_ at a dosage of 2 ml/kg b.wt. twice a week for 1 month, and then treated with sorafenib at a dosage of 10 mg/kg b.wt. daily after 15 days, reveals that the hepatic lobule seems rather normal. (**d**) A liver micrograph of a rat that was given a dose of 60 mg/kg b.wt. of DEN and then administered 2 ml/kg b.wt. of CCl_4_ twice a week for 1 month. Afterward, the rat was treated with ZnO-SPION-Ag nanocomposite for 15 days. The hepatic lobule appeared to be relatively normal. (**e**) A liver micrograph of a rat treated with 60 mg/kg b.wt. of DEN and subsequently given 2 ml/kg b.wt. of CCl_4_ twice a week for 1 month, followed by ZnO-SPION-Ag nanocomposite loaded with sorafenib for 15 days, reveals congestion in the portal tract and hepatocytes that appear mostly normal.

The histological analysis of liver slices from the control group shows the presence of normal hepatocytes arranged in a radial pattern originating from the central vein. Figure 8a demonstrates that hepatocytes possess vesicular nuclei, with some being binucleated, and sinusoids separating the strands of liver cells. Rats were given a dosage of 60 mg/kg b.wt. of DEN, followed by a dose of 2 ml/kg b.wt. of CCl_4_ twice a week for 1 month. After this treatment, tumor cells resembling pseudo-glands, fibrotic sheets, and hydropic degeneration were seen in Fig. 8b. Rats were induced for HCC then treated by sorafenib showed the hepatic lobules looked to be normal as shown in Fig. 8c. Rats were induced for HCC then treated with ZnO-SPION-Ag nanocomposite showed both the hepatic lobules and the portal tracts appeared to be normal as seen in Fig. 8d. Rats were induced for HCC then treated with ZnO-SPION-Ag nanocomposite loaded with sorafenib showed both in the normal hepatic lobules and portal tracts as demonstrated in Fig. 8e.

## Discussion

Diethyl nitrosamine is a frequent initiator of HCC, while CCl_4_ is used to increase the severity of carcinogenesis. In the rat, HCC develops as a result of the production of alkyl DNA-DEN adducts and the triggering of numerous nuclear abnormalities by DEN. DEN is a well-known hepatocarcinogenic substance that has been utilized in animal experiments^42^. In rats, DEN/CCl_4_ treatment proved successful in causing HCC^27^. Sorafenib is costly and correlated with adverse events (AEs). Furthermore, some treated individuals do not respond to the treatment^43^. Its efficacy is severely limited due to its restricted bioavailability caused by its low solubility in water and its quick elimination and metabolism^44^. It also causes skin toxicity, diarrhea, hypertension, and hand foot syndrome^22,23^. Several forms of stimuli sensitive sorafenib nano-delivery systems have been developed and thoroughly tested in order to overcome the mentioned disadvantages and acquire the desired sorafenib delivery system with significant clinical benefits^45,46^. These smart sorafenib nano-delivery systems have many advantages over conventional formulations, including increased targetability and bioavailability, managed drug release, and tumor accumulation capability^47^. In the present study, we use the drug delivery system to load the sorafenib on ZnO-SPION-Ag nanocomposite which contain Ag NPs and ZnO NPs that have anticancer effect and SPION nanoparticles to improve the targeting of the drug to hepatocellular carcinoma in male albino rats with low dose and no side effects. DTL (denticle less protein homolog), commonly known as CDT2, is a gene that contains numerous WD40-repeat domains and is important in controlling CDT1 degradation following DNA damage. Cell division is an important phase in tumor growth because it results in the formation of two comparable clones, which is important for tumor growth and carcinogenesis. DTL (CDT2), which is often coupled with the cullin ring E3 ubiquitin ligase (CRL) to create a complex (CRLCDT2), primarily plays a crucial role in genomic stability inside tumor cells, which is required for tumor cells to replicate. In the present study, the results of positive control group higher than that of the negative control group that agree with Zuyin Li et al.^48^ study that demonstrate that DTL expression is substantially higher in tumor tissues than in non-tumor tissues. After treatment, significant down-regulation in the DTL gene in all studied groups that agree with the study of Rana et al.^19^. Our results revealed a highly significant drop in DTL level in the sorafenib group compared to the positive control group, which is consistent with Wilhelm et al.^49^. Also, at p value 0.001, the ZnO-SPION-Ag NPs group (Nano group) showed a highly significant decrease in DTL level when compared to the positive control group. Our findings were consistent with those of Zhang et al., who discovered that nanoparticles or nano matrix could improve antitumor activity in vivo^50^. At p value 0.001, the drug loading group (Composite group) also showed a highly significant decrease in DTL level compared to the positive control group. These findings were consistent with those of Sheng et al., who discovered that sorafenib nanoparticles inhibit tumor growth in vivo^51^. DUSP1 has been shown to be involved in cell cycle inhibition, apoptosis, and senescence^24^. A South Korean study found that DUSP1 acted as a tumor suppressor during hepatocarcinogenesis and that DUSP1 expression was linked to p53 activation. By creating a heterodimer with NF-κB and blocking its translocation to the nucleus, NFKBIA suppresses NF-κB. A recent study investigated the frequency of NFKBIA genotype and haplotype polymorphism distribution between HCC and control tissues. It has been observed that NFKBIA expression in liver cancer tissue is lower than in normal tissue, and that negative NFKBIA expression predicts poor prognosis in individuals with primary HCC^24^. SOCS2 expression has also been shown to be significantly decreased in HCC and to be related with aggressive tumor development and a poor prognosis in HCC patients^24^. The results of the positive control group in this research were lower than those of the negative control group, which agrees with the findings of Meng et al.^24^. In conclusion, DTL, DUSP1, NFKBIA, and SOCS2 were found to be closely related to HCC and may give significant information for improving HCC treatment and prognosis. Our results showed that the anticancer effect of the nanocomposite loaded with sorafenib better than sorafenib drug or nanocomposite separated. In the present study, it was confirmed that DTL expression was upregulated in HCC, and DUSP1, NFKBIA and SOCS_2_ were downregulated according to Meng et al. study^24^ and opposite effect with using the treatments. When compared to the control group, the efficiency of DEN/CCl_4_ in producing liver dysfunction as evaluated by raised AST and ALT. Multiple studies have found support for this increase, which is caused by leakage from injured or necrotic cells, and it may be utilized as evidence for HCC formation in rats intoxicated with DEN^27^ that agree with our study and by using sorafenib loaded on nanocomposite lower the activates more better than using sorafenib or nanocomposite alone. The substantial rise in ALP as a marker of DEN/CCl_4_ liver toxicity, on the other hand, can be due to bile duct mechanical blockage, inability to eliminate the enzyme, and hence its accumulation in the blood^52^. The decrease in ALP activity following drug delivery system therapy might be due to a reduction in mechanical blockage in the bile duct. Lipid peroxides, quantified as MDA, are extensively used as a prominent indicator of oxidative stress in individuals with liver injury to evaluate the extent of oxidative damage. Higher hepatic NO generation plays critical functions in neoplastic transformation and progression via DNA damage. When compared to controls, the oxidative toxic impact of DEN/CCl_4_ can easily be recognized as a substantial increase in MDA and NO, as well as a considerable decrease in catalase, GPx, SOD, and GST as important antioxidant enzymes significantly essential for the scavenging of MDA as a marker of oxidative stress. This is supported by Zhang et al.'s recent DEN/CCl_4_ research^53^ who observed that DEN may cause HCC by interacting with critical macromolecules such as antioxidant enzymes, DNA, lipids, and DNA repairing system enzymes. Furthermore, it is widely accepted that CCl_4_ biotransformation by cytochrome P-450 results in trichloromethyl free radical (CCl_3_*) and trichloromethyl proxy free radical (CCl_3_OO*) as two metabolites associated with ROS generation, lipid peroxidation, and a decrease in CAT, SOD, GST, and GPx enzymatic activities^54^. Furthermore, the obtained findings are consistent with previous research of Hussein and Khalifa, as well as Kadasa et al.^55^ They observed a substantial decrease in antioxidant enzyme activity and relative gene expression in DEN-induced rats as compared to controls. The current study also found a substantial increase in antioxidant enzymes (CAT, SOD, GST, and GPx) following administration of nanocomposite loaded with sorafenib or nanocomposite alone against sorafenib as the conventional therapy for HCC. ROS have been shown to be a direct source of somatic cell mutagenesis and a cancer promoter, hence they are classified as a life-threatening and oncogenes product^27^. According to Hassan et al. AFP was significantly raised in the group that was induced for HCC in the present study^56^ and decreased by using different treatments sorafenib, nanocomposite and nanocomposite loaded with sorafenib. From the previous results we achieved for our aim and, we found that the nanocomposite ZnO-SPION-Ag itself has anticancer effect and by loading sorafenib we improve the anticancer effect of the drug with low dose and improve the anticancer effect of the ZnO-SPION-Ag nanocomposite.

## Conclusion

Drug delivery for sorafenib has been achieved through loading of it on a novel synthetic ecofriendly nanocomposite (ZnO-SPION-Ag NPs), loading drug has a promising improving of anticancer effect of the sorafenib without side effects due to using biological method for preparation eco-friendly nanocomposite with attractive role in targeting sorafenib for cancer cells with no resistance and low dose. DTL, DUSP1, NFKBIA and SOCS2 genes closely related to the degree of pathogenicity of hepatocellular carcinoma and giving a good valuable insights about the progress of HCC and prognosis and this in turn prove our suggestion in the role of sorafenib targeting hepatocellular carcinoma by loading on the ecofriendly synthetic nanocomposite and by the comparison was made between the group treated with ZnO-SPION-Ag nanocomposite and the positive control group, it was approved that it has excellent anticancer effect on hepatocellular carcinoma effect.

## Data Availability

The data presented in this study are available on request from the corresponding author.
